# Interobserver variability in the delineation of the tumour bed using seroma and surgical clips based on 4DCT scan for external-beam partial breast irradiation

**DOI:** 10.1186/s13014-015-0370-3

**Published:** 2015-03-13

**Authors:** Bing Guo, Jianbin Li, Wei Wang, Min Xu, Qian Shao, Yingjie Zhang, Chaoqian Liang, Yanluan Guo

**Affiliations:** Department of Radiation Oncology, Shandong Cancer Hospital and Institute, Jiyan Road, Jinan, Shandong Province 250117 China; Medicine and Life Sciences College of Shandong Academy of Medical Sciences, Jinan University, Jinan, Shandong Province People’s Republic of China

**Keywords:** Breast cancer, Four-dimensional computed tomography, Interobserver variability, Surgical clips, Seroma

## Abstract

**Background:**

To explore the interobserver variability in the delineation of the tumour bed using seroma and surgical clips based on the four-dimensional computed tomography (4DCT) scan for external-beam partial breast irradiation (EB-PBI) during free breathing.

**Methods:**

Patients with a seroma clarity score (SCS) 3 ~ 5 and ≥5 surgical clips in the lumpectomy cavity after breast-conserving surgery who were recruited for EB-PBI underwent 4DCT simulation. Based on the ten sets of 4DCT images acquired, the tumour bed formed using the clips, the seroma, and both the clips and seroma (defined as TB_C_, TB_S_ and TB_C+S_, respectively) were delineated by five radiation oncologists using specific guidelines. The following parameters were calculated to analyse interobserver variability: volume of the tumour bed (TB_C_, TB_S_, TB_C+S_), coefficient of variation (COV_C_, COV_S_, COV_C+S_), and matching degree (MD_C_, MD_S_, MD_C+S_).

**Results:**

The interobserver variability for TB_C_ and TB_C+S_ and for COV_C_ and COV_C+S_ were statistically significant (p = 0.021, 0.008, 0.002, 0.015). No significant difference was observed for TB_S_ and COV_S_ (p = 0.867, 0.061). Significant differences in interobserver variability were observed for MD_C_ vs MD_S_, MD_C_ vs MD_C+S_, MD_S_ vs MD_C+S_ (p = 0.000, 0.032, 0.008), the interobserver variability of MD_S_ was smaller than that of MD_C_ and MD_C+S_ (MD_S_ > MD_C+S_ > MD_C_).

**Conclusions:**

When the SCS was 3 ~ 5 points and the number of surgical clips was ≥5, interobserver variability was minimal for the delineation of the tumour bed based on seroma.

## Background

Breast-conserving therapy (BCT), which involves a wide local excision followed by radiotherapy to the whole breast, is the standard treatment for early-stage breast cancer [1]. The efficacy of BCT for the treatment of early-stage breast carcinoma has been established in multiple randomised trials [2,3]. External-beam partial breast irradiation (EB-PBI) has recently garnered increasing interest [4,5]. Several studies reported that EB-PBI, which delivers radiotherapy to the postoperative tumour bed (TB) with a margin of adjacent breast tissue, could achieve excellent results in certain patients. However, there are inherent challenges in defining accurate target volumes for partial breast irradiation (PBI). Studies demonstrate significant interobserver variability between radiation oncologists in defining the lumpectomy cavity, indicating the need to improve the accuracy and consistency in the delineation of the TB [6,7].

Surgical clips and seroma are important markers for delineating the TB for EB-PBI [7,8]. Kirby et al. [7] reported that the number of implanted markers influences the accuracy of target delineation and that five to six surgical clips are preferable for TB delineation for PBI or breast boost radiotherapy. Landis et al. [8] indicated that the shift of the centre of mass (COM) decreased and the percent volume overlap (PVO) increased significantly as the seroma clarity score (SCS) increased. The influence of the number of metal clips, SCS, delineation experience and contouring guidelines for the delineation of TB in CT images have been investigated [6,7,9-11]. However, the effect of interobserver variability on the delineation of the TB using seroma and surgical clips based on 4DCT scan is not clearly established. To investigate the impact of different markers on interobserver variability in the delineation of TB based on 4DCT scan for EB-PBI, we analysed the TB delineated by five observers in this study based on clips, seroma, and both clips and seroma.

## Methods

### Patients

Twenty patients who underwent wide-local excision of breast cancer with full-thickness unstitching of the excision cavity (10 left-sided and 10 right-sided lesions) followed by EB-PBI between June 2009 and November 2013 were included in this study. To improve the delineation accuracy and consistency, all of the enrolled patients had SCS 3 ~ 5 and ≥5 surgical clips to mark the boundaries of the lumpectomy cavity. For every patient, five or more roundish surgical clips with diameters of 2 mm were implanted. The surgical clips were fixed to the superior, inferior, medial, lateral, and posterior walls of the surgical cavity, respectively (median number: 6) [12]. The average interval from lumpectomy to 4DCT scan was 10 weeks (range, 3-16 weeks). All patients were free of chronic lung diseases, and their ventilation functions were normal. Written informed consent was obtained from all patients with the approval of the Institutional Review Board (Shandong Tumour Hospital Ethics Committee).

### Four-dimensional CT image acquisition

All twenty patients were immobilised in the supine position on a breast board using an arm support (with both arms above the head to adequately expose the breast). 4DCT images and respiratory signals were acquired with a thickness of 3 mm at the conclusion of the standard CT simulation using a 16-slice Brilliance Big Bore CT scanner (Philips Medical Systems, Inc., Cleveland, OH, USA). The signals were sent to the scanner to label a time tag on each CT image. GE Advantage 4D software (General Electric Healthcare, Waukesha, WI, USA) sorted the reconstructed 4DCT images into 10 respiratory phases based on these tags, with 0% corresponding to end inhalation (EI) and 50% corresponding to end exhalation (EE). Then, the constructed 4DCT image sets were transferred to the Eclipse treatment planning system (Eclipse™ 8.6; Varian Medical Systems, Palo Alto, CA) for structure delineation.

### Observers

Five observers specialising in radiation treatment of breast carcinoma with more than five years of radiotherapy experience performed the delineations.

### Tumour bed delineation

**Figure 1:**
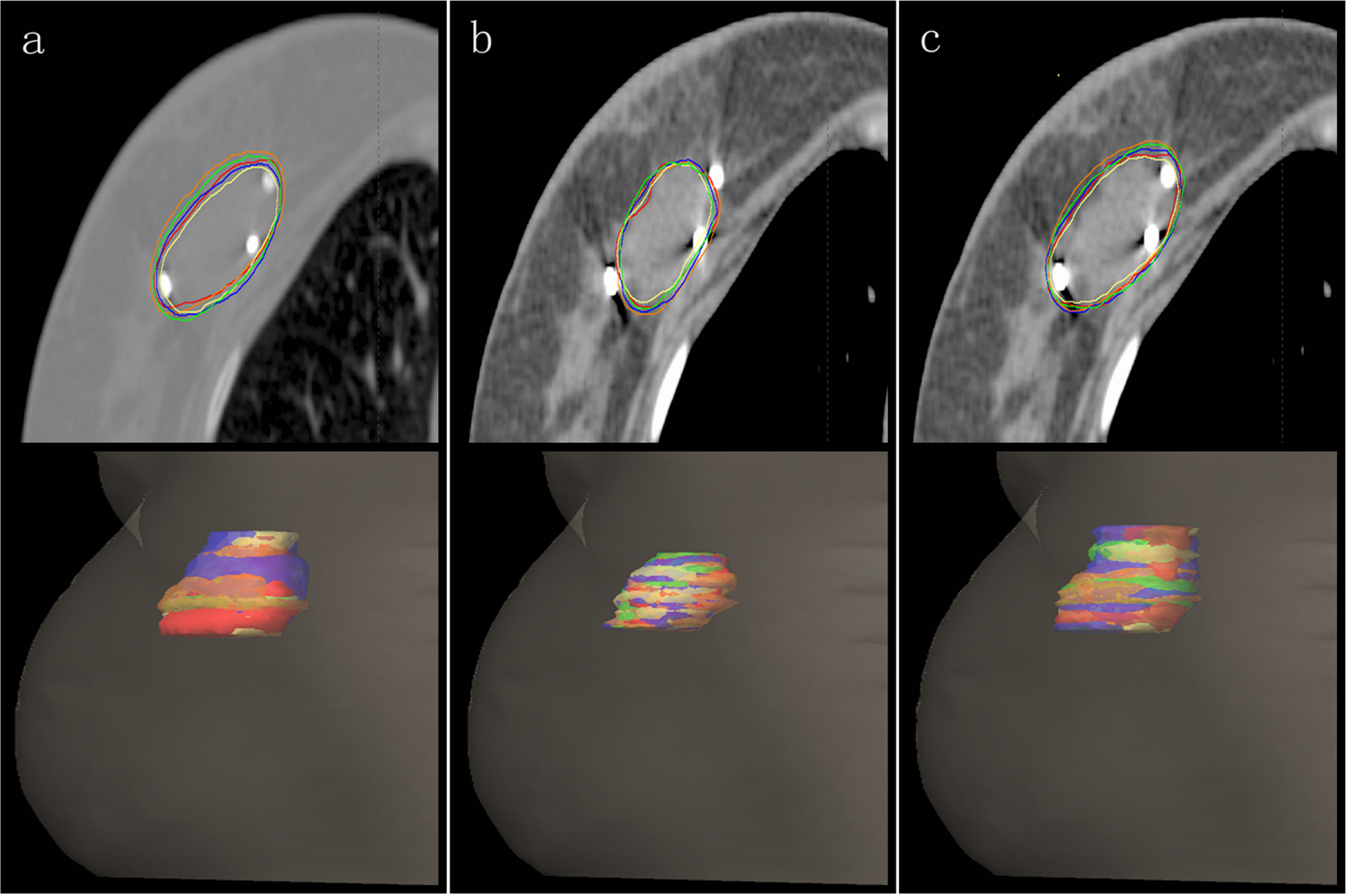
**Single CT slice and volumetric image of one patient with delineated TB**_**C**_ **(a), TB**_**S**_ **(b) and TB**_**C+S**_ **(c) of all 5 observers**. TB_C_, the TB delineated based on clips; TB_S_, the TB delineated based on the seroma; TB_C+S_, the TB delineated based on both seroma and clips.

The 10% ~ 90% phases of the 4DCT images were registered on the 0% phase images, which served as the basic phase image. The tumour beds were delineated from the ten sets of 4DCT images based on the clips, the seroma, and both the clips and seroma (termed TB_C_, TB_S_, TB_C+S_, respectively) according to a set of guidelines [13] (Figure 1). When the TB was delineated based on clips, we adjusted the window level and width to minimise the impact of seroma for contouring. All observers outlined a single test case that was reviewed prior to commencing the study to ensure that the guidelines were being followed.

### Observation parameters

**Figure 2:**
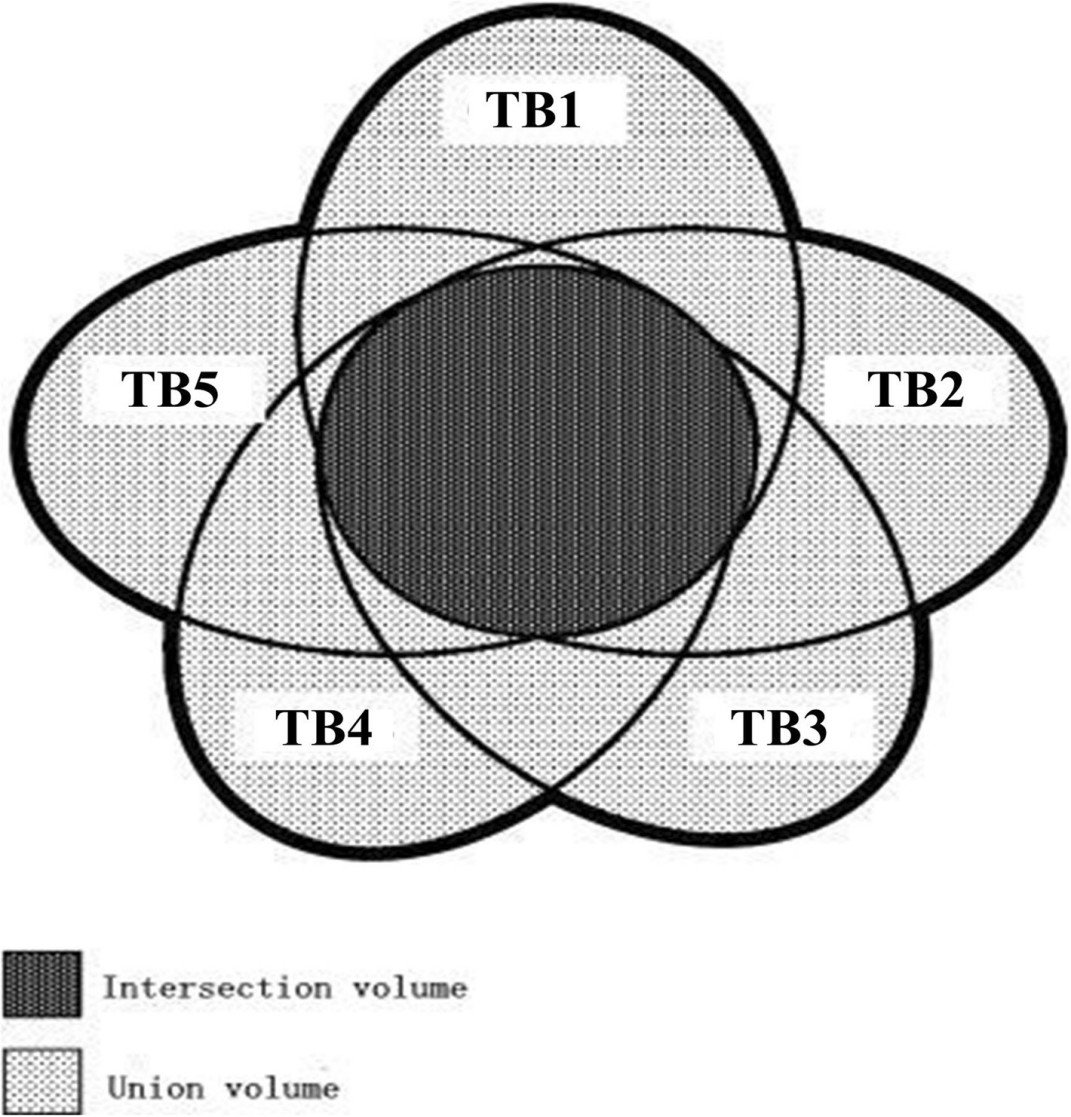
**Mismatch between the TB delineated by five observers**. The union volume is defined as the union of the five tumour beds, whereas the intersection volume is the intersection between the five tumour beds.

To quantify interobserver variability, the following parameters were calculated: the volume of TB (TB_C_, TB_S_, TB_C+S_), which was an average value generated from the ten contours of the registered images for each patient, and the interobserver coefficient of variation (COV_C_, COV_S_, COV_C+S_) for each patient. The COV was defined as the ratio between the standard deviation and the average volume of TB. For TB_C_, TB_S_, and TB_C+S_, an evaluation of the matching degree among the various observers in the EE phase was also performed. For each patient, the ratio between the intersection volume (the intersection among the volumes delineated by the five observers) and the union volume (the union among the volumes delineated by the five observers) was calculated (termed MD_C_, MD_S_ and MD_C+S_, respectively) [14] (Figure 2). The volume variability between the clips, the seroma, and both the clips and seroma based on the same observer was also calculated.

### Statistical analysis

SPSS 19.0 software was used for statistical analyses. A normal distribution test and a test for homogeneity of variance were performed. To investigate the interobserver variability in the delineation of the TB based on different makers in 4DCT images, a univariate analysis of variance (ANOVA) was used to compare differences in the TB (TB_C_, TB_S_, TB_C+S_), COV (COV_C_, COV_S_, COV_C+S_) and MD (MD_C_, MD_S_, MD_C+S_) between observers. The volume variability between the TB_C_, TB_S_ and TB_C+S_ was also compared using ANOVA. P < 0.05 was considered significant.

## Results

### TB volume

The volumes of TB_C_, TB_S_ and TB_C+S_ are listed in Table 1. The average volumes of TB_C,_ TB_S_ and TB_C+S_ were 21.98 cc (range 8.70-45.13), 14.36 cc (range 3.14-41.80), and 28.79 cc (range 12.88-55.80), respectively. The volume variability between the TB_C_, TB_S_ and TB_C+S_ was statistically significant (p < 0.05) (Table 2). Furthermore, the average duration from lumpectomy to 4DCT scan was 10 weeks (range, 3-16). The volume of TB_C_ was similar to that of TB_S_ for six patients during weeks 4-8, the TB_C_ was less than TB_S_ in two patients during weeks 0-3, and the TB_C_ was larger than TB_S_ for twelve patients during weeks 8-16.

**Table 1:** **The volumes of TB**_**C**_ **, TB**_**S**_ **and TB**_**C+S**_ **(cc)**

**Patients**	**TB**_**C**_	**TB**_**S**_	**TB**_**C+S**_
1	27.62(21.40-35.58)	10.28(9.16-11.09)	31.28(21.97-41.28)
2	30.20(24.22-32.13)	13.80(12.34-15.27)	32.58(24.93-36.33)
3	29.79(26.69-34.52)	24.01(21.33-25.93)	40.28(34.96-47.32)
4	45.13(42.98-47.03)	41.80(38.63-45.81)	55.80(48.73-67.71)
5	27.39(22.80-32.17)	14.48(12.38-19.49)	33.33(26.43-41.73)
6	24.94(20.59-31.99)	32.48(30.30-37.82)	47.61(44.20-59.24)
7	8.70(8.24-9.12)	6.40(5.60-8.13)	15.87(11.54-21.91)
8	16.91(14.17-22.80)	15.62(11.16-20.84)	19.14(17.55-20.01)
9	17.36(14.09-23.07)	13.03(10.77-16.64)	22.57(17.29-29.60)
10	19.43(16.62-24.45)	27.46(24.15-29.68)	40.21(34.14-47.77)
11	25.15(22.44-30.97)	14.46(13.08-17.61)	28.43(23.49-35.69)
12	24.26(21.78-30.37)	25.20(24.21-26.71)	33.87(31.53-40.34)
13	24.39(21.66-31.57)	8.38(6.58-11.50)	29.41(23.05-36.77)
14	15.02(11.93-23.00)	4.73(3.37-7.38)	17.64(12.88-24.60)
15	14.82(8.40-22.39)	8.22(6.26-9.25)	19.44(14.92-25.81)
16	16.89(13.07-26.14)	5.67(4.12-7.84)	20.09(12.57-28.15)
17	23.22(11.32-33.70)	4.44(3.89-4.97)	30.11(25.58-35.88)
18	15.68(12.31-22.72)	6.55(4.6-9.12)	19.19(13.37-25.41)
19	10.84(7.51-18.40)	3.14(2.49-4.5)	12.88(9.44-19.88)
20	21.83(16.64-28.61)	7.13(5.97-8.75)	26.16(20.08-33.18)
Mean	21.98	14.36	28.79

*Abbreviations*: TB_C_, the tumour bed delineated based on clips; TB_S_, the tumour bed delineated based on the seroma; TB_C+S_, the tumour bed delineated based on both seroma and clips.

**Table 2:** **The interobserver variability for TB**_**C**_ **, TB**_**S**_ **and TB**_**C+S**_ **(cc,Mean ± SD)**

	**TB**_**C**_	**TB**_**S**_	**TB**_**C+S**_	**F**	**P**
observer 1	19.87 ± 9.14	14.16 ± 11.27	25.94 ± 12.19	5.798	0.005
observer 2	21.06 ± 8.75	14.26 ± 10.97	25.57 ± 10.76	6.815	0.002
observer 3	20.01 ± 7.8	12.78 ± 9.89	24.88 ± 10.43	8.312	0.001
observer 4	28.34 ± 6.91	14.07 ± 11.74	34.16 ± 11.13	19.565	0.000
observer 5	20.61 ± 9.07	16.55 ± 10.42	33.41 ± 13.80	12.001	0.000
F	3.644	0.315	3.053		
P	0.008	0.867	0.021		

*Abbreviations*: TB_C_, the tumour bed delineated based on clips; TB_S_, the tumour bed delineated based on the seroma; TB_C+S_, the tumour bed delineated based on both seroma and clips.

The interobserver variability for TB_C_, TB_S_ and TB_C+S_ is listed in Table 2. The interobserver variability for TB_C_ and TB_C+S_ was statistically significant (p = 0.021, 0.008). However, the interobserver variability for TB_S_ was not statistically significant (p = 0.867).

### COV

The interobserver variability for COV is listed in Table 3. The interobserver variability for COV_C_ and COV_C+S_ was statistically significant (p = 0.002, 0.015), but the interobserver variability for COV_S_ was not statistically significant (p = 0.061).

**Table 3:** **The interobserver variability for COV (%,Mean ± SD)**

	**COV**_**C**_	**COV**_**S**_	**COV**_**C+S**_
observer 1	4.08 ± 2.49	4.3 ± 2.13	3.81 ± 2.41
observer 2	4.04 ± 1.33	3.83 ± 2.04	3.23 ± 0.71
observer 3	4.24 ± 1.45	5.31 ± 2.85	4.61 ± 2.1
observer 4	2.66 ± 1.42	4.83 ± 2.30	2.69 ± 1.05
observer 5	5.10 ± 2.17	3.42 ± 1.55	3.52 ± 1.90
F	4.591	2.331	3.332
P	0.002	0.061	0.015

*Abbreviations*: COV_C_, coefficients of variability formed by TB_C_; COV_S_, coefficients of variability formed by TB_S_; COV_C+S_, coefficients of variability formed by TB_C+S_.

### MD

Table 4 lists the differences in MD between the volumes delineated based on clips, seroma, and both clips and seroma in the EE phase. The interobserver variability for the MD_C_, MD_S_ and MD_C+S_ was statistically significant (F = 16.866, p = 0.000). There were also significant differences between MD_C_ and MD_S_, MD_C_ and MD_C+S_, and MD_S_ and MD_C+S_ (p = 0.000, 0.032, 0.008); the interobserver variability for MD_S_ was smaller than that of MD_C_ and MD_C+S_ (MD_S_ > MD_C+S_ > MD_C_).

**Table 4:** **The differences in MD between the volumes delineated based on clips, seroma and both clips and seroma**

**patients**	**MD**_**c**_	**MD**_**s**_	**MD**_**c+s**_
1	0.30	0.41	0.29
2	0.37	0.51	0.39
3	0.37	0.45	0.39
4	0.31	0.69	0.49
5	0.38	0.46	0.39
6	0.38	0.58	0.37
7	0.40	0.44	0.30
8	0.34	0.46	0.49
9	0.36	0.50	0.34
10	0.36	0.63	0.46
11	0.43	0.52	0.40
12	0.45	0.59	0.51
13	0.37	0.37	0.39
14	0.29	0.43	0.30
15	0.32	0.61	0.44
16	0.31	0.41	0.39
17	0.18	0.31	0.55
18	0.24	0.48	0.42
19	0.25	0.30	0.34
20	0.27	0.63	0.45
$\overline{x}$±s	0.33 ± 0.07	0.49 ± 0.11	0.40 ± 0.07

*Abbreviations*: MD_C_, the ratio between the intersection volume and the union volume based on clips; MD_S_, the ratio between the intersection volume and the union volume based on seroma; MD_C+S_, the ratio between the intersection volume and the union volume based on both clips and seroma.

## Discussion

The accuracy of target volume delineation is critical for EB-PBI. The optimal target volume for EB-PBI remains to be established. Most reports define the tumour excision cavity or postoperative seroma as the target volume in treatment planning [7,8,15]. However, these studies also reported significant interobserver variation in delineating post-lumpectomy cavities. van Mourik et al. [16] investigated breast target volume delineations among thirteen observers in eight patients. They reported that the presence of clips or seroma reduced interobserver variability but that significant volumetric and spatial interobserver variability was observed in clinical target volume (CTV) even with the help of delineation guidelines. Therefore, reducing interobserver variability is a pressing issue. To improve delineation accuracy and consistency, we selected patients with SCS 3 ~ 5 and ≥5 surgical clips to mark the boundaries of the lumpectomy cavity.

Seroma clarity and volume in the lumpectomy cavity decreased as a function of time from surgery to the CT scan. The use of the CT-based seroma to guide the EB-PBI target volume is difficult due to a lack of clearly defined standards. Kader et al. [6] selected 205 women with early-stage breast cancer to undergo planning CT after breast conserving surgery and found that the mean seroma volume decreased from 47 cc to 30 cc during postoperative weeks 3-8, stabilised during weeks 9-14 (mean 21 cc) and was involuted beyond 14 weeks (mean 9 cc). In our study, the average duration from lumpectomy to 4DCT scan was 10 weeks (range, 3-16). The volume of TB_C_ approached the volume of TB_S_ for six patients during weeks 4-8, and the TB_C_ < TB_S_ in two patients during weeks 0-3. However, after 8 weeks, the volume of TB_C_ was larger than that of TB_S_ for 12 patients. Therefore, given the magnitude and time trends of seroma volume and clarity loss, the optimal time to obtain the planning CT scan for PBI is within 8 weeks after surgery.

Surgical clips are not always consistent with the edge of seroma and the boundary of the lumpectomy cavity [13,17]. Ding et al [13] measured the three dimensional displacements of the GTV_C_, the GTVs and the GTV_C+S_ and found that in the LR, AP and SI directions, the displacements were 0.9 mm, 1.05 mm and 1.20 mm for GTV_C_; 0.80 mm, 1.05 mm and 0.80 mm for GTVs; and 0.90 mm, 1.20 mm and 1.40 mm for GTV_C+S_, respectively. In other words, the three dimensional displacements of GTV_C+S_ were greater than those of GTV_C_ and GTV_S_. Yang et al. [17] also measured the distance between surgical clips and the edge of the seroma in a coronal plane in women who underwent wide local excision of breast cancer and reported that the mean seroma edge extended beyond the clips by 0.3-0.5 cm. This study indicates that the volume of TB_C+S_ delineated by observers was significantly larger than TB_C_ and TB_S_. These results may be due to decreased seroma clarity and volume in the lumpectomy cavity from the time of lumpectomy to the 4DCT scan, as well as the variability between the TB_C_, TB_S_, TB_C+S_ regarding treatment margin.

Interestingly, we observed no significant differences between observers for TB_S_ (p = 0.867) and COV_S_ (p = 0.061). This could be explained by the short average duration from lumpectomy to planning CT and because the SCS 3 ~ 5 in the lumpectomy cavity improved the visualisation of the surgical cavity. Landis et al. [8] reported similar results in patients of SCS 4 and 5, as the average COM shift was 3 mm and 2 mm, respectively, and the PVO was 77% and 87%, respectively. Wong et al. [18] also reported that after reviewing contouring guidelines, the differences in seroma target volume (STV), CTV, and planning target volume (PTV) were no longer statistically significant. Although Dzhugashvili et al. [9] reported that the conformity index of TB delineation was significantly improved by the placement of surgical clips within the lumpectomy cavity, a higher level of interobserver concordance was observed by the five observers when the SCS was 3 ~ 5 points in the lumpectomy cavity. These data suggest that interobserver variability in the delineation of TB based on seroma was not obvious when the SCS was 3 ~ 5 points.

Although interobserver variability in the delineation of the TB based on seroma was minimal, the clinical reality could vary across regions. This was a retrospective study, and all the enrolled patients had undergone wide-local excision of breast cancer with full-thickness unstitching of the excision cavity. Full thickness closure of the excision cavity and oncoplastic surgical procedures are becoming more widely practiced in some regions, and these surgical methods could reduce the rate of seroma and its reliability as a marker of the TB. Therefore, patients should be given five or more surgical clips to reduce interobserver variability when only clips are used in clinical practice. Moreover, the placement of clips in the surgical cavity could be based on guidelines, and the duration from lumpectomy to 4DCT scan could be shortened.

Hurkmans et al. [19] reported that intra- and, to a greater extent, interobserver variability in the delineation of breast target volume on CT scans can be large. Both Dzhugashvili et al. [20] and Yang et al. [21] also reported interobserver variability (similar to the results presented here) in the delineation of the TB based on clips. These results can be explained by the fact that tissue stranding from the surgical cavity, proximity to muscle, dense breast parenchyma, and benign calcifications may be mistaken for surgical clips. Moreover, limited soft-tissue contrast on CT makes it an unreliable modality for detecting a layer of the image lacking surgical clips and when distinguishing between surgically induced densities and normal glandular breast tissue. Finally, the experience of the radiation oncologist and subjective determination of the location of the post-surgical cavity contribute to interobserver variability. Using a combination of information to more precisely define the TB, such as surgical reports, clinical palpation of the surgical defect and CT-based planning, may decrease interobserver variability. Additionally, clear communication between the surgeon and radiotherapist, including diagrammatic explanations, are crucial for accurately targeting the TB.

Cover et al. [22] reported that when the EE phase was reviewed in the sagittal plane, gating would reduce the mean tumour mobility from 6.3 ± 2.0 mm to 1.4 ± 0.5 mm. Moreover, 4DCT simulation scan can reduce motion artefacts [23,24]. Therefore, to investigate spatial mismatches of interobserver variability in the delineation of the TB based on different markers, the difference between the MD_C_, MD_S_ and MD_C+S_ were further analysed and compared based on the end-exhalation phase. Our study found interobserver variability between the MD_C_, MD_S_ and MD_C+S_, as well as between the MDc and MDs, MD_C_ and MD_C+S_, and MD_S_ and MD_C+S_. Our results suggest spatial mismatch existed among observers in the delineation of the TB based on clips, the seroma, both the clips and seroma. Additionally, the MD of the seroma was larger than that of the clips as well as both the clips and seroma, and the MD of both the clips and seroma was larger than that of the clips alone (Table 4). However, the average CI reported by van Mourik et al. [16] was 0.53, which was considerably higher than that in the present study. This difference is likely due to differing target volume and calculation methods. Landis et al. [8] and Li et al. [25] reported even higher CI values ranging from 0.73-0.75; however this analysis focused on the PTV instead of the TB, the CI of which increases due to the larger volumes.

Image-guided techniques can improve clip and seroma localisation during treatment, potentially enabling the use of a smaller GTV-to-PTV margin. Ultrasound image guidance has also been investigated [26]. However, it is unclear if the margin is sufficient to account for interobserver contour variability. Further studies are needed to determine, whether contouring variability could result in an underdosing of the clips or seroma. Thus, it is imperative that future studies aiming to reduce margins from current treatment practice take interobserver contour variability into consideration.

## Conclusions

The results of our study suggest that interobserver variability is smaller in the 4DCT delineation of the TB based on seroma compared with clips or both clips and seroma when the SCS was 3 ~ 5 points and the number of surgical clips was ≥5 in the lumpectomy cavity. Interobserver volume differences were observed between the TBs delineated based on surgical clips, and those delineated based on both the clips and seroma. This was also the case for spatial mismatch (MD), which was measured and analysed at the end-expiration phase. Optimising the time from lumpectomy to 4DCT scan is necessary to minimise interobserver variability in the delineation of the TB. If the time from lumpectomy to 4DCT simulation scan could be appropriately chosen, the delineation of the target volume based on seroma may be more reasonable in radiotherapy treatment planning.

## Abbreviations

BCT: Breast-conserving therapy
PBI: Partial breast irradiation
EB-PBI: External-beam partial breast irradiation
SCS: Seroma clarity score
4DCT: Four-dimensional computed tomography
COV: Coefficient of variation
MD: Matching degree
COM: Centre of mass
PVO: Percent volume overlap
EI: End- inhalation
EE: End-exhalation
